# Classification of Beef *longissimus thoracis* Muscle Tenderness Using Hyperspectral Imaging and Chemometrics

**DOI:** 10.3390/foods11193105

**Published:** 2022-10-06

**Authors:** Sara León-Ecay, Ainara López-Maestresalas, María Teresa Murillo-Arbizu, María José Beriain, José Antonio Mendizabal, Silvia Arazuri, Carmen Jarén, Phillip D. Bass, Michael J. Colle, David García, Miguel Romano-Moreno, Kizkitza Insausti

**Affiliations:** 1 IS-FOOD (Institute of Innovation and Sustainable Development in Food Chain), Department of Agricultural Engineering, Biotechnology and Food, Campus de Arrosadia, UPNA (Universidad Pública de Navarra), 31006 Pamplona, Spain; 2 IS-FOOD (Institute of Innovation and Sustainable Development in Food Chain), Department of Engineering, Campus de Arrosadia, UPNA (Universidad Pública de Navarra), 31006 Pamplona, Spain; 3 Department of Animal, Veterinary, and Food Sciences, University of Idaho, Moscow, ID 83844, USA; 4 Lev2050, Polígono Industrial Plazaola, Manzana E, nave 10, 31195 Aizoáin, Spain

**Keywords:** meat quality, texture, HSI, PLS-DA, chemometrics

## Abstract

Nowadays, the meat industry requires non-destructive, sustainable, and rapid methods that can provide objective and accurate quality assessment with little human intervention. Therefore, the present research aimed to create a model that can classify beef samples from *longissimus thoracis* muscle according to their tenderness degree based on hyperspectral imaging (HSI). In order to obtain different textures, two main strategies were used: (a) aging type (wet and dry aging with or without starters) and (b) aging times (0, 7, 13, 21, and 27 days). Categorization into two groups was carried out for further chemometric analysis, encompassing group 1 (n_group1_ = 30) with samples with WBSF ˂ 53 N whereas group 2 (n_group2_ = 28) comprised samples with WBSF values ≥ 53 N. Then, classification models were created by applying the partial least squares discriminant analysis (PLS-DA) method. The best results were achieved by combining the following pre-processing algorithms: 1st derivative + mean center, reaching 70.83% of correctly classified (CC) samples and 67.14% for cross validation (CV) and prediction, respectively. In general, it can be concluded that HSI technology combined with chemometrics has the potential to differentiate and classify meat samples according to their textural characteristics.

## 1. Introduction

Meat is commonly defined by its compositional quality, which includes the lean/fat ratio and a group of attributes known as meat palatability [1]. Subsequently, the appearance, odor, firmness, juiciness, tenderness, and, finally, flavor are considered, among others. Meat tenderness is outlined as one of the most influential organoleptic characteristic when evaluating beef quality [2], and it is important to bear in mind that meat tenderness can be modified by both intrinsic and extrinsic factors, including pre-slaughter and postmortem mechanisms [3]. Other meat quality parameters that have a direct impact on meat tenderness, which are also subjected to change, include pH, intramuscular fat (IMF), and total and soluble collagen content.

Due to the importance that tenderness presents when referring to consumer satisfaction, special postharvest techniques have been carried out throughout history by the meat industry to improve the mentioned attributes and, as a result, give an added value to the final product [4]. For instance, Bowker et al. [5] used electrical stimulation for this purpose while the effect of techniques such as tenderstretch, which is characterized by hanging the beef carcasses from the pelvis, on tenderness has also been studied [6]. Other methods such as blade tenderization [7,8], high-temperature conditioning [9], and the use of ultrasound [10] have been used to increase meat tenderness.

One of the most common practices is aging [11,12,13,14,15], also known as “ripening” or “conditioning”, which takes place between the slaughter of the animal and the final consumption of the meat. Furthermore, it is defined as a postmortem technique performed under controlled conditions to improve several traits of the obtained meat [16]. There is debate about how long it should last; the temperature, HR%, and air flow; and the benefits and/or the possible drawbacks of this technique. Moreover, aging can be wet (under vacuum packaging) or dry (leaving the meat samples to age without any packaging). Furthermore, the introduction of starters in meat products is becoming increasingly more common to enhance the desired final characteristics [17,18].

Techniques based on imaging and spectroscopy are considered promising methods for assessing and classifying meat quality [19,20]. Hyperspectral imaging (HSI) is an emergent technology that can both provide internal information of meat and spatially locate it [21]. In addition, the introduction of chemometrics is required to process the information. Note that the term *chemometrics* was defined by Massart et al. [22] as “the chemical discipline that uses mathematical, statistical, and other methods employing formal logic to (a) design or select optimal measurement procedures and experiments, and (b) to provide maximum relevant chemical information by analyzing chemical data.”

As the meat industry pursues rapid meat quality assessment tools that require less human intervention, and provide data in the most objective and accurate means, non-invasive techniques are becoming more prominent [23]. It is valued that HSI is both an accurate and non-destructive procedure, which makes it very appropriate for meat sample analyses. Being able to predict the eating quality of meat is a key point in maintaining the competitiveness of the meat industry [24].

Regarding the recent applications of vibrational spectroscopy, a number of previous studies have identified HSI as a means of assessing the chemical, textural, and structural characteristics of meat [25,26,27,28,29,30]. Until then, attempts to classify meat samples into unbalanced groups as far as the number of steaks is concerned, the use of mean spectra to carry out the process, or efforts to predict quality parameters were observed. However, more research is needed to bolster the use of HSI as a quality assessment tool in meat. Therefore, the objective of the present research was to move one step forward and create a tenderness classification model by applying HSI combined with chemometrics to beef samples under the Protected Geographical Indication (PGI) “Ternera de Navarra”. Moreover, the novelty in visualizing the tenderness distribution maps on the steaks was shown.

## 2. Materials and Methods

### 2.1. Sample Collection and Preparation

Twelve intact bulls, which were reared under the Protected Geographic Indication “Ternera de Navarra”, were used in this study. After the standard fattening period for the PGI established in the EEC 1483/2004 [31], from 6 to 13 months of age, the animals were transported to the abattoir the day before slaughter in compliance with the current European Community of Animal Welfare in transport. They were stunned with a captive bolt and subsequently slaughtered and dressed according to the specifications outlined in the EEC 93/119/1993 [32].

**Figure 1:**
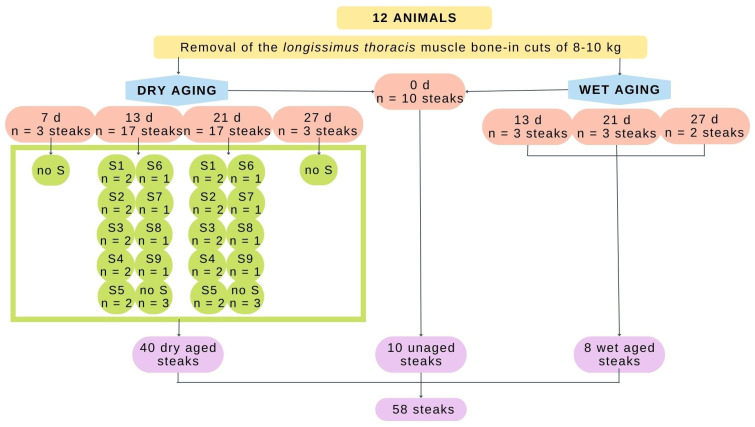
Distribution of the samples utilized for this study according to the type of aging method, time, and starter used. S1, S2, S3, S4, S5, S6, S7, S8, and S9 represent the dissimilar starters and “no S” refers to non-inoculated beef.

Then, 24 h postmortem, the *longissimus thoracis* muscles were removed from both sides of the carcass in 8-10 kg bone-in cuts, equivalent to the short loin and sirloin portions. The samples were transported under refrigeration from the Almameat S.L.U. abattoir (EU-licensed commercial abattoir) located in Salinas de Pamplona, Spain, to the meat quality laboratory located within the School of Agricultural Engineering and Biosciences-UPNA, also in Pamplona, Spain. Once in the lab, the bone-in cuts were assigned to different aging processes (dry/wet aging with or without starters) and periods (0, 7, 13, 21, and 27 days) to obtain different final textures. After, they were further cut into smaller portions, providing a total of 58 meat samples, as shown in Figure 1.

The dry-aged samples were aged under the same conditions in 2 cold storage units (Kide, Berritua, Bizkaia, Spain) with a controlled temperature of 8 °C and a relative humidity of 85%. Regarding the former, the purpose of selecting such a temperature was to take refrigeration to the extreme in order to enhance microbial growth since at 4 °C, the recommended temperature for dry aging, such growth is slowed down. Moreover, the air in the chambers was renewed twice a day for 15 min.

Starter cultures were used to enhance the aging effect of part of the dry-aged meat cuts. Nine starters were applied: five bacteria both superficially and intramuscularly, two fungi superficially, and two yeasts intramuscularly (patent number: P202130774).

For all samples, once the aging days elapsed, pH was measured. Then, the meat cuts were sliced, a 20-mm-thick (±0.2 mm) steak was taken for intramuscular and collagen analysis, and a 25-mm-thick steak was separated for the textural analysis Warner Bratzler shear force (WBSF), in which the maximum force is measured, showing the hardness of meat. All samples were then vacuum packaged and frozen at −18 °C (±2) until analysis.

### 2.2. Reference Measurements

Prior to tenderness evaluation, the samples were thawed at 4 °C for 24 h. Samples were cooked on a water bath by immersion (J.P Selecta S.A., Abdera, Barcelona, Spain) according to the AMSA guidelines [33] until they reached an internal temperature of 70 °C. The internal temperature was monitored at the center of the steaks using a probe Digitron (Hereford, UK) model 3246 thermocouples. For texture analysis, a TA-XT2i Stable Micro Systems texturometer was used connected to an IBM-compatible Foxen computer, with a microprocessor AutenticAMD-K6 (tm) 3D processor. The software employed was “Texture Expert” version 1.22 to Windows (Stable Micro Systems, Surrey, UK).

The maximum shear force was assessed using the above mentioned texturometer system equipped with a stainless-steel Warner Bratzler V Slot Blade probe (Stable Micro Systems, Surrey, UK) with a 1.06 mm thickness containing a vee-shaped (60° angle) cutting edge, with a capacity of 30 kg at a velocity of 1.70 mm/s, complying with the specifications of this method [34]. System force calibration was carried out with a 5 kg weight. Between six and eight replicates per sample were analyzed with the following dimensions: 1 cm^2^ (square cross-section), with the muscle fibers parallel to the longitudinal axis of the sample, and at least 30 mm long. As a result, the software provides the resistance to shear in Newtons (N), also known as the characteristic toughness of meat. It should be noted that, prior to texture analysis, raw samples were first scanned to obtain the hyperspectral images in order to further proceed with the destructive texture measurement. The registered WBSF values were used to categorize the steaks into two homogeneous groups of tenderness.

Sample pH was measured using a pH-meter (Crison GLP 22, Hach Lange Spain S.LU., Barcelona, Spain), introducing the penetration electrode in different positions of the *longissimus thoracis* muscle and ensuring a homogeneous data acquirement. Two measurements were taken for each steak to finally calculate the average value. Among the different samples, the electrode was cleaned with distilled water. Furthermore, the intramuscular fat content was measured in duplicate for each sample using the ISO 1443:1973 [35] protocol.

Total and soluble collagen were assessed by applying the methodology proposed by Bonnet and Kopp [36], based on the hydrolysis of the proteins present in the meat samples both in acid and heat media.

#### Statistical Analyses

Factorial analysis principal component analysis (PCA), using the principal component extraction and Varimax rotation method [37], was used to assess how the meat samples behaved regarding the measured attributes. The basis of this employed method lies in the creation of linear combinations of the data known as principal components (PCs), which can be used to summarize the data with minimal loss of information. The software used to accomplish the analyses was RStudio 3.3.2. (RStudio, PBC, Boston, MA, USA). Analysis of variance (ANOVA) was carried out with the same software, which was applied with the aim of identifying the statistical differences among the established groups.

### 2.3. Image Acquisition

**Figure 2:**
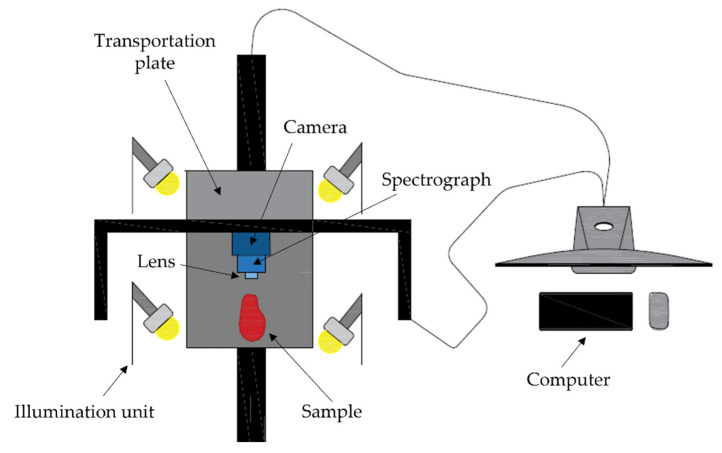
Schematic representation of the components that constitute the hyperspectral imaging system.

In total, 58 beef steaks from *longissimus thoracis* were scanned on one side using a setup for hyperspectral imaging consisting of (Figure 2): (a) a transportation plate to move the sample, (b) an illumination unit composed of 4 28 W halogen lamps, (c) a hyperspectral camera, and (d) a computer equipped with the software Xeneth 2.5. and ACT Controller for the camera and transportation plate control, respectively. As for the hyperspectral camera used (Xenics, Model Xeva-1.7- 320, Leuven, Belgium), it was coupled to a spectrograph (ImSpector N17E, Specim, Spectral Imaging Ltd., Oulu, Finland) sensitive in the NIR region from 900 to 1700 nm and a lens (Xenics, Model OPT-000107, SWIR 16 mm f/1.4, Leuven, Belgium) with a focal length of 16 mm. The resolution of the system was 320 × 256 pixels.

This image acquisition system has a line scanning or pushbroom configuration, which acquires a whole line of an image, requiring the sample to be moved under the field of view of the camera each time. In order to satisfactorily acquire the images, the distance between the sample and the camera was set to a 40 cm height and the platform speed reached 7 mm/s. Regarding the integration time, it was established in 2000 µs. Due to this configuration, the images presented the following size: 320 columns (0.75 mm/pixel), 256 λ (every 3.14 nm, approximately), and a variable number of rows according to the sample size.

### 2.4. Image Processing

**Figure 3:**
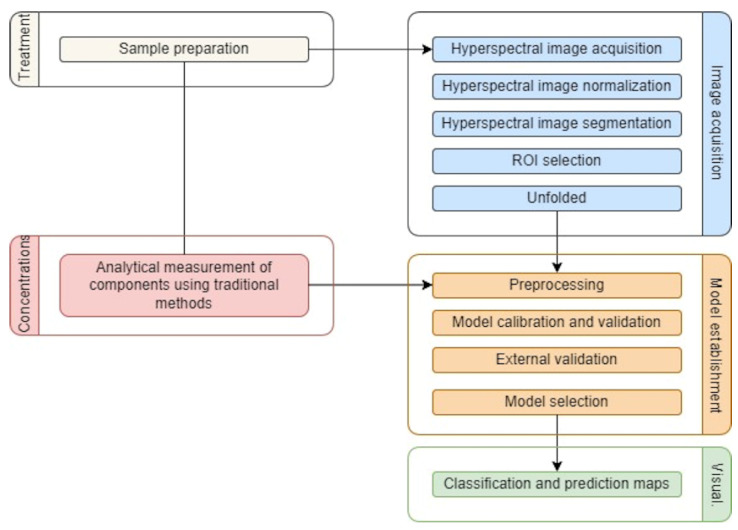
General flowchart of the image process carried out. Adapted from Ma et al. [38].

The processing of the images was carried out with the PLS_Toolbox version 9.0 (Eigenvector Research Inc., Wenatchee, WA, USA) under MATLAB R2020a (The MathWorks, Natick, MA, USA). Prior to the classifications, data must be correctly prepared by considering several issues, as determined in Figure 3 and explained as follows.

#### 2.4.1. Normalization

The first step in image processing consists in the normalization of the raw reflectance values obtained (I_raw_) to values between 0 and 1. For this, two references of the maximum and minimum reflectance, also called white (I_white_) and black (I_black_), are needed. The black image is related to the minimum reference value, which was obtained by taking images with the camera lens covered and the lights turned off. Concerning the white, which is also known as the maximum reference, it is associated with the upper limit. For this purpose, images were taken from a *Spectralon* tile (Standard 99%, Labsphere) with the following dimensions: 600 × 50 × 10 mm. Then, the corrected image (I_c_) was calculated by applying Equation (1):

(1) $$I_{c}=\frac{I_{\mathrm{raw}}{-I}_{\mathrm{black}}}{I_{\mathrm{white}}{-I}_{\mathrm{black}}}$$

#### 2.4.2. Dead Pixel and Spike Removal

Dead pixels are caused due to the appearance of irregularities in the detectors. These are defined as single isolated pixels placed randomly in spatial positions consequently derived from black pixels [39]. Vidal and Amigo [40] reported that approximately 1% of pixels can be treated as dead pixels when NIR detectors are used for HSI. Consequently, in the present case, the mentioned pixels were located to subsequently interpolate their values with the neighboring ones. For this, starting from the image at each wavelength, a 2-D interpolation was performed. Using the built-in function “scatteredInterpolant” of MATLAB, a value was assigned to the dead pixels according to their neighbors.

Concerning the possible spikes, they are related to false intensity peaks, which should be removed [40]. In this study, for each pixel, the mean reflectance was calculated and any value ±6 times the standard deviation was considered a spike to which the mean value of the two nearest neighbors was assigned.

#### 2.4.3. Segmentation and Region of Interest (ROI)

The geometry of the samples in the acquisition of the images should be considered due to the nature of the cameras. Square images are often captured; so, in case the sample does not cover all the scanned area, the background must be removed [40]. This background is characterized by highly noisy spectra that could affect the results.

**Figure 4:**
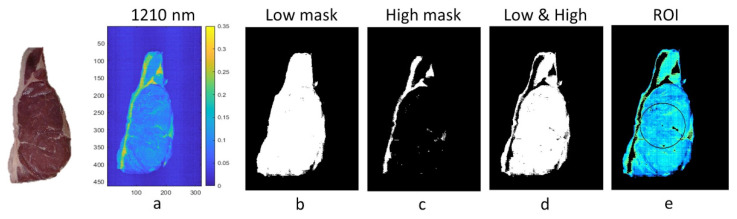
Low and high masks of a meat sample: (**a**) the image in band 1210 nm was used to select the threshold values; (**b**) image after applying the low mask; (**c**) image after applying the high mask; (**d**) image after combining the low and high masks; (**e**) inner ring as the region of interest for the calibration dataset.

In this study, two masks, namely low and high, were used to remove the background and fat in each image because, as reported by Cluff et al. [26], pixels from the fat could negatively affect the final performance of the models. The image at 1210 nm (Figure 4a) was selected to segment the background and the fat. The selected region in the meat to which the low mask was applied is illustrated in Figure 4b. Figure 4c shows the fat selected using the high mask, which was further removed. Figure 4d represents the selected region of each steak after combining both masks, that is, removing the background and the fat. The threshold values used for the low and high mask were 0.068 and 0.19, respectively, that is, all pixels with reflectance values between 0.068 and 0.19 were selected for further analysis.

As for the region of interest, a ROI must be selected from the segmented image that has undergone the dead pixel and spike removal process. In this analysis, and due to the nature of the experiment, an inner ring was selected that covered 25% of the sample (Figure 4e), counting from the central point. It should be noted that this ROI was only selected for the samples belonging to the calibration set. Thus, the initial number of pixels was considerably reduced to approximately 350,000 pixels. Subsequently, the computational limitations were overcome.

However, the meat cuts used for the external validation/prediction (Pred) were used in their entirety (Figure 4d). This means that once the models were built, classification maps were obtained to visualize the texture distribution throughout the sample.

#### 2.4.4. Unfolding

The principal objective of the unfolded process is to decompose the three-dimensional hypercube into a two-dimensional matrix in which each row represents the spectrum of one pixel [41]. In other words, it aims to rearrange the spectra from the hypercube with three dimensions ((a) rows, (b) columns, and (c) wavelengths) to a two-dimensional matrix ((a) rows x columns against (b) wavelengths) [42]. As a case in point, the first 33 wavelengths were subtracted from the matrix to avoid data tempering.

#### 2.4.5. Pre-Processing of the Data

Moreover, once the matrixes are prepared, they are pre-processed, with the purpose of eluding unwanted effects over the spectral appraising [40]. Among the types of pre-processing methods that could be applied, the following stand out: (a) spectral smoothing techniques (SM), (b) standard normal variate (SNV), (c) mean center (MC), and (d) first-(1st der) and second-order (2nd der) derivatives.

Regarding the first one, the Savitzky–Golay (SG) de-noising method of a polynomial order of a 2- and 15-point window was used. It is characterized by polynomial filtering [43]. Meanwhile, it is depicted as being ideal due to its capacity to minimize the least squares error by matching the polynomial to the outline of the data set-up as noisy [44]. Indeed, it has the ability to smooth and differentiate absorption spectra simultaneously even though the fact that makes SG filtering so interesting is the unambiguous and clear way in which the filtering is executed [45].

In the scatter correction category, the SNV technique is widely applied for the purpose of disbanded minimization and correct baseline shift, whose basis is to provide the entire spectrum with a common scale [46]. For the mentioned purpose, each individual spectrum is centered for subsequent division by their standard deviation [47], as shown in Equation (2):

(2) $${\mathrm{xSNV}}_{i,j}=\frac{\left(x_{i,j}-{\bar{x}}_{i}\right)}{\sqrt{\frac{\sum_{j=1}^{p}{\left(x_{i,j}-{\bar{x}}_{i}\right)}^{2}}{p-1}}}$$

where xSNV_i,j_ is the transformed spectrum and x_i,j_ is the original element of the spectrum i at variable j, ${\bar{x}}_{i}$ is the mean of spectrum i, and p is the number of wavelengths.

Regarding the mean center pre-processing technique, it can reduce the systematic noise. For this purpose, the average value of each variable is firstly calculated and then subtracted from the data [48].

On the other hand, spectral derivatives can be found. In the current study, first- and second-order derivatives were implemented. Both treatments remove baseline offsets in the data and improve the apparent spectral features [49], whereas the latter, apart from the mentioned advantages, also separates overlapping peaks [50]. The most common algorithm employed for this is the above-explained SG. Indeed, derivative spectroscopy is focused on the calculation of the derivatives starting from the original spectrum with regard to the variables and their use for further detection of the absorption bands [45].

In the present case, the models were constructed by combining the explained pre-processing techniques to evaluate the performance of the data.

### 2.5. Multivariate Data Analysis

The last step in the pre-processing phase is multivariate analysis, which can be applied for either information extraction, regression, or classification from the hyperspectral data. The main applications are focused on material recognition and target detection, among others [51]. For this purpose, the relationships among the spectrum and the physical and chemical composition of the samples are established. In this concrete case, mathematical and statistical algorithms are developed to process the data [52]. As an example, the detection, interpretation, and elimination of possible outliers is an essential phase in multivariate analysis.

#### 2.5.1. Supervised Analysis (PLS-DA)

Furthermore, the statistical analysis called partial least squares-discriminant analysis (PLS-DA) was applied, which belongs to the supervised analytical techniques category. A remarkable fact is that PLS-DA is categorized as one of the most employed statistical techniques in the area of chemometrics [53]. Its main applications are found in food authentication, medical diagnoses, and in the field of the forensic sciences [54].

In this case, orthogonal linear combinations of the original variables are established, which are called latent variables (LVs) or PLS components [55]. These combinations, in addition to allowing graphical visualization and making the model more clearly understood, set up the maximum covariance among the X and Y variables. As a case in point, the Y-block of a PLS-DA analysis is characterized by describing which objects are included in the classes of interest. Thus, in the Y matrix, membership is represented by assigning 1 to the samples that fit the class and assigning 0 to the ones that do not belong to the mentioned group [56]. In the present study, for the PLS-DA model, two groups were included regarding the values obtained for the reference measurements.

Noteworthy, the implemented method, before completing the algorithmic model, transforms the high-dimensional data into vectors as a prerequisite. Indeed, it can estimate whether one of the samples belongs to a defined group according to the categories of the selected variables [57].

#### 2.5.2. Model Validation and Accuracy

In order to guarantee the reliability of the model, the samples were randomly split into calibration and validation datasets. The percentage retained for calibration was 70% for each tenderness group established, whereas the remainders were used to externally test the models. Therefore, of the 58 meat steaks, 42 samples made up the calibration block while the 16 remnants constituted the test dataset.

To evaluate the attainment of the models, dissimilar parameters are taken into consideration, such as the ones listed below: (a) the confusion matrix and table, which finally determine the percentage of correctly classified samples (% CC); (b) the explained variance; and (c) the sensitivity and specificity values. According to Ortega et al. [58], sensitivity is related to the test facility satisfactorily recognizing a condition or division whereas specificity is related to the ability to exclude it. Subsequently, when the sensitivity and specificity approach 1, the performance of the model is expected to be better. To calculate these values, Equations (3) and (4) should be applied [59]:

(3) $$\mathrm{SEN}=\frac{\mathrm{TP}}{\mathrm{TP}+\mathrm{FN}}$$

(4) $$\mathrm{SPE}=\frac{\mathrm{TN}}{\mathrm{TN}+\mathrm{FP}}$$

where TP (true positive) is the number of samples classified in group 1; FN (false negative) is the number of samples wrongly classified as group 1; TN (true negative) is the number of samples correctly classified in group 2; and, finally, FP (false positive) is the number of samples incorrectly identified as group 2.

Thus, the overall accuracy of the model, explained by the % CC, was calculated by executing the following Equation (5) [59]:

(5) $$\%\mathrm{CC}=\frac{\mathrm{Confusion}\;\mathrm{matrix}\;\mathrm{main}\;\mathrm{diagonal}}{\mathrm{Total}\;\mathrm{number}\;\mathrm{of}\;\mathrm{predictions}}$$

However, all the models were likewise cross validated (CV), which is included in the calibration procedure. This consisted of the removal of data from the calibration group and validation of the model with the same data used for calibration. The method employed for cross validation was the Venetian blind with the subsequent settings: 10 splits and a maximum of 20 LVs.

## 3. Results and Discussion

### 3.1. Descriptive Analysis

Table 1 reports the differences between the groups regarding the tenderness (*p* ˂ 0.001).

The WBSF values ranged from 34.68 to 106.10 N. Based on this, two categories (group 1 < 53 N; group 2 ≥ 53 N) were used for further hyperspectral imaging analysis, with the objective of classifying the samples according to their WBSF values (n_Group 1_ = 30 and n_Group 2_ = 28). It is worth mentioning that most of the samples used in the present study were identified as tough according to the AMSA guideline [33] since they exceeded the established intermediate limit of 46 N. Regardless, the aim of this study was not distinguish between tender and tough meat cuts but to demonstrate the ability of HSI to differentiate groups of different tenderness. The key point, and the novelty of this study, is that both textural categories comprise a similar number of steaks whereas in previous research, this was not the case as far as we are concerned.

According to the four remaining parameters, no statistically significant differences were observed (*p* > 0.05). The pH means were similar for both groups. However, the soluble and total collagen and intramuscular fat tended to be lower for the more tender samples. In order to facilitate the interpretation of the mentioned results, a PCA analysis was carried out as shown in Table 2.

**Figure 5:**
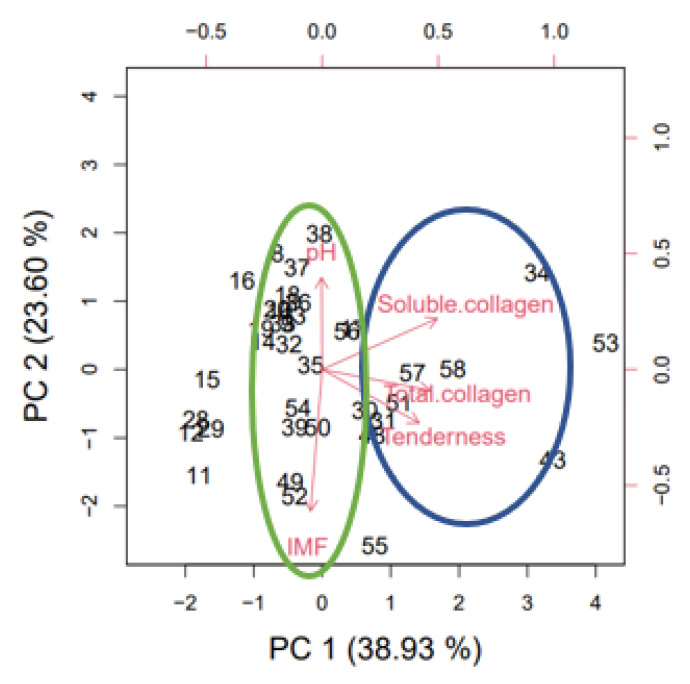
Results obtained from the PCA carried out with Rstudio for the meat attributes under study.

As a case in point, the importance of each principal component is shown. The main fact is that the first 4 PCs explain 94.16% of the variance. To visualize the results obtained, in Figure 5, it can be observed how in this case, the first principal component related the tenderness and total and soluble collagen parameters so that WBSF increased with the collagen content.

In this line, and according to Li et al. [60], the literature presents antagonistic findings regarding the relationship among collagen features and tenderness properties. Subsequently, it was reported that WBSF is not a good indicator of connective tissue strength, which may explain the weak correlation between collagen and tenderness. Moreover, Nishimura [61] reported that even though many researchers have attempted to correlate intramuscular fat connective tissue (IMCT) with tenderness via either organoleptic evaluation or mechanical testing, no conclusive results were obtained. Meanwhile, Sentandreu [62] defined IMCT and collagen concentration as the “background toughness” of samples. There are scientists who deny that collagen may affect texture [63]. However, Torrescano et al. [64] found a high positive correlation between insoluble collagen and WBSF of raw beef. Collagen is an abundant connective tissue protein in muscle. Its molecules are bound together through intermolecular crosslinks that help provide structure and strength, having an important function in meat texture. However, as showed in the metanalysis performed by Li et al. [60], further in-depth studies are required in order to obtain a positive or negative correlation of this meat parameter with the obtained tenderness.

On the other hand, the principal component 2 set other differences and an inversely proportional relationship between pH and IMF was established. Specifically, when the intramuscular fat content decreased, pH increased. It is worth mentioning that IMF, according to Hocquette et al. [65], is the outcome obtained from the balance of the uptake, synthesis, and degradation of triacylglycerols.

Regarding pH, the measures taken 24 h postmortem are used in abattoirs for quality control due to the influence of ultimate pH (pHu) on the final meat tenderness. Lomiwes et al. [66] determined beef *longissimus* tenderness in relation to the pHu values. In the present study, pHu values were below 5.6 (data not shown). The mentioned authors concluded that the toughest beef samples were obtained when the pHu data ranged from 5.79 up to 6.2. In this line, there are proteins that play an important role, such as calpains, and a lack of such proteins will result in less tender meat [67]. Silva et al. [68] reported that the cooking temperature of meat influences the relationship among ultimate pH and tenderness, concluding that medium cooking temperatures of about 65 °C result in a curvilinear relationship between the said parameters.

### 3.2. Spectra Interpretation

**Figure 6:**
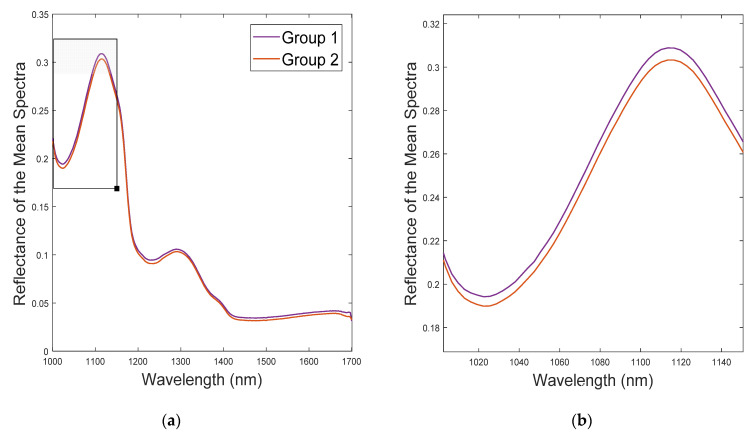
Reflectance of the mean spectra of the two groups determined according to their WBSF values: (**a**) represents the full spectrum (1000–1700 nm) captured by the NIR-HSI camera employed; (**b**) zoom of the 1010–1460 nm area.

Figure 6a represents the mean spectra of the two determined categories in reflectance mode in the 1000–1700 nm spectral range.

As can be observed, both groups maintained the same trend along the spectrum. The major difference was in the magnitude of the reflectance, where group 1 registered slightly higher values than those acquired in group 2. A greater separation between groups is observed in the 1000 to 1100 nm range (Figure 6b). Both groups 1 and 2 are characterized by two molecular vibrations, with peaks occurring at the following wavelengths: 1113 and 1289 nm. Peaks around 1250 nm are associated with the second stretching overtone of C-H bonds, which is, in turn, associated with the presence of fatty acids [69,70]. According to Pieszczek et al. [70], particular bands can be used to identify the presence of chemical components. For instance, proteins, which are correlated with the N-H bond, were identified due to a highlighted absorption band around approximately 1187 nm [71].

### 3.3. PLS-DA Models

Hyperspectral imaging analysis, combined with chemometrics, is mainly based on the pre-processing and preparation of data to proceed with the creation of models thanks to the application of methods such as PLS-DA. For the present case, dissimilar PLS-DA models were built by testing different pre-processing algorithms, although only the three best results are shown. Therefore, to differentiate a model that meets the required specifications for its subsequent application, their appraisal was evaluated first by % CC of the two tenderness groups. Table 3 shows the obtained outcomes for the best three PLS-DA models in terms of % CC. As a way to interpret its results, it should be considered that the percentages placed in the diagonal of the matrix are correctly classified whereas the remainders are misclassified.

The% CC values for CV ranged from 66.57 to 74.58%. If the models were externally predicted (Pred), the mentioned% CC decreased, from 54.79 to 74.67%. If the results were analyzed individually, the model to which a pre-processing of 1st der + MC was applied registered the best approach, with an average % CC for both groups of CV and Pred of 70.83 and 67.14%, respectively, selecting 12 LVs and with an explained variance of about 99.70%. On the other hand, if, instead of the above-mentioned pre-processing, SM + 2nd der + MC were considered (11 LVs and 99.55% of the explained variance), the average value for CV was of 67.65% while for Pred, it was reduced by 1.37 points, reaching a value of 66.28%. Similar behavior was detected for the SM + SNV + MC model (12 LVs and 100% of the explained variance), where the CV and Pred values, on average, were 70.11 and 64.35% for the two tenderness groups, respectively.

The sensitivity and specificity values were used to evaluate the performance of the models and are listed in Table 4. As a reminder, as the values approach 1, the prediction of the model is expected to be better. Remarkably, as shown by % CC, higher values were achieved by the 1st der + MC model. When comparing the mean values, the results obtained for the Pred set were, on average, lower than the trained ones.

Balage et al. [25] published a PLS-DA model, concerning the textural data, with sensitivity values of 61%, 62%, and 68% in calibration, CV, and Pred, respectively. If these values are compared to the ones obtained in the present research, it can be seen how in the latter, the sensitivity data is somewhat superior since it reached 70.85% and 67.15% in CV and Pred, respectively. Furthermore, they chose to test two groups of beef samples, with WBSF = 45 N as the boundary between the two of them. The above-mentioned authors selected a ROI, which corresponded to the core position from which the WBSF value was obtained. Balage et al. [72] determined that tougher meat tend to absorb more light in comparison to more tender samples.

Cluff et al. [26] used a line scan HSI system to develop a method for classifying beef according to the shear force (SSF) values. For this purpose, they considered samples from the *longissimus* with SSF ≥ 206 N (n = 26) as tough whereas SSF < 206 N (n = 446) was determined as tender. Moreover, Cluff et al. [26] carried out some trials to identify how the presence of fat could alter the final grading percentages. When fat was not removed from the optical scattering images, the discriminant model presented % CC of 70% for the training dataset while the validation block registered 75% of the correctly classified pixels. When comparing their data to the achieved results in the present study, % CC is quite similar for CV, being 0.83% higher for the proposed study, whereas % CC for Pred is about 7.87% larger in their research.

Other authors such as Naganathan et al. [29] opted for a non-homogeneous distribution of the samples among the tenderness groups. In this concrete case, they developed a canonical discriminant model (CDA) using three tenderness categories: (a) SSF ≤ 205.80 N, (b) 205.80 N < SSF < 254.80 N, and (c) SSF ≥ 254.80 N. Therefore, from the 111 meat samples, 94 were included in the tender group, 12 were identified as intermediate, and 5 as tough samples. They also selected a ROI of 200 × 600 pixels starting from the center of the slice. Concerning the overall accuracy, they obtained a 96.40% accuracy for CV. When comparing their results with the ones obtained in the case under study, the key point lies in the distribution of the samples. They opted for an unbalanced allotment while the present research was carried out with the most homogeneous categorization as possible.

Regarding beef meat and its quality attributes, Reis et al. [30] evaluated the use of HSI and chemometrics to assess the chemical, textural, and structural characteristics of meat. In their research, they concluded that with the acquired data, they were able to identify the chemical composition of meat. Naganathan et al. [28] studied how to forecast beef texture characteristics with three-dimensional chemometric analysis of hyperspectral images. They affirmed that the Fisher’s linear discriminant (FLD) method demonstrated potential to discriminate among samples with dissimilar textures. However, they opted for a non-homogeneous distribution of the samples since the first category included 173 samples whereas the second included only 40 steaks.

Recently, Jiang et al. [27] tried to classify fresh broiler fillets according to their texture. They grouped the slices into least, moderate, and very tender, obtaining % CC of about 66% for the training set whereas 56% was obtained for the test array, selecting 5 LVs in this case. Subsequently, the percentages obtained in the present case (1st der + MC) for beef meat classify 6.82% and about 16.58% better than with their models for CV and Pred, respectively.

#### 3.3.1. Tenderness Distribution Maps

**Figure 7:**
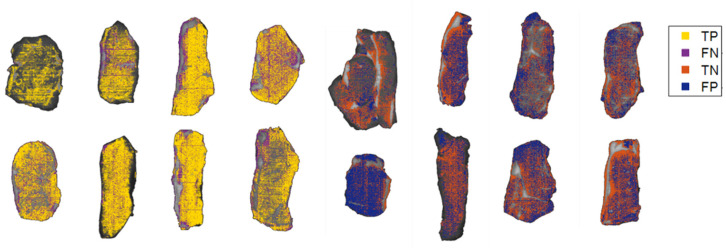
Tenderness distribution maps of the external validation samples after classification (1st der + MC). The steaks on the first four columns represent the samples used to test group 1 whereas the remainders gather the ones employed to validate the group 2. TP = true positive; FN = false negative; TN = true negative; FP = false positive.

With the aim of facilitating the interpretation of the attained outcomes, it is possible to graphically visualize the classifications pixel by pixel in the samples used for external prediction. Thus, the spatial distribution of tenderness was observed by creating classification maps of the samples. As each pixel was classified within the two tenderness groups established, in Figure 7, the yellowish color represents the pixels known as true positives whereas purple represents FN of group 1. With regard to group 2, TN is depicted in orange while pixels painted in blue portray FP. As a reminder, TP is the number of samples classified in group 1, FN is the number of samples wrongly classified as group 1, TN is the number of samples correctly classified in group 2, and, finally, FP is the number of samples incorrectly identified as group 2.

#### 3.3.2. VIP Score Plot

**Figure 8:**
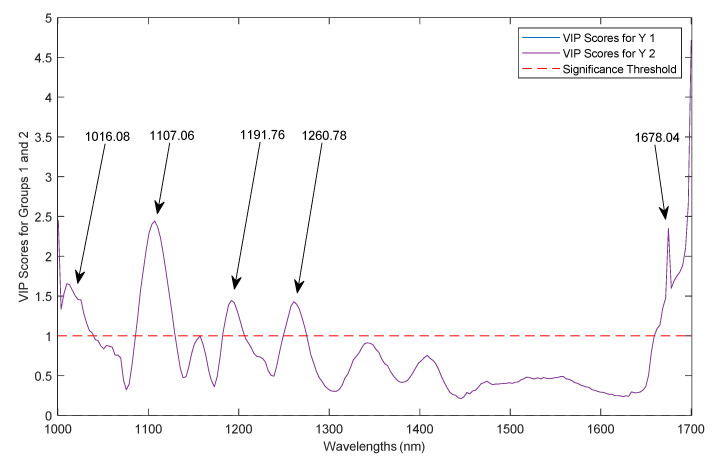
VIP scores for the two classification categories.

Moreover, there are wavelengths that present higher weights in both CV and prediction. Hence, there is a parameter known as variable importance in projection (VIP) that scores the impact of these wavelengths. Specifically, the higher the VIP score, the more important the response variable is as a predictor. If the VIP scores are greater than 1.0, these wavelengths are classified as highly influential whereas those with VIP scores lower than 0.8 are insignificant [41]. In this line, it can be said that all wavelengths above a threshold of 1.0 are considered as optimal for further classification processes. Therefore, Figure 8 shows the VIP score plot for the 1st der + MC model from which the classifications for the established groups 1 and 2 were made.

Five wavelengths were observed for which the graded values were above the determined threshold: (a) 1016.08, (b) 1107.06, (c) 1191.76, (d) 1260.78, and (e) 1678.04 nm. The VIP bands ranging between 1100 and 1400 nm are associated with the second overtone region where C-H plays an important role. More specifically, these vibrations are caused by the stretching of the radicals C-H, C-H_2_, and C-H_3_ [73]. The 1600 nm region is also associated with N-H stretching vibration [74]. Hence, the wavelengths previously associated with proteins and fatty acids are stated as being influential in the classification models built.

## 4. Conclusions

In the present study, the suitability of hyperspectral imaging combined with chemometrics to distinguish beef samples from *longissimus thoracis* muscle according to tenderness was tested.

In accordance with the results obtained from PCA, pH and IMF presented an inversely proportional relationship whereas collagen and WBSF were directly related.

Furthermore, regarding the multivariate analysis, it was shown that reducing the systematic noise by MC and removing baseline offsets by 1st der gave the best classification results, with % CC reaching 70.83 and 67.14% for CV and Pred, respectively. Consequently, it can be stated that hyperspectral imaging combined with chemometrics has the potential to discriminate among different meat textures.

Regarding the classification maps of the samples involved in the external validation block, it can be concluded that in the 1st der + MC model, 73.05% of the pixels were correctly classified in the established tenderness group 1 whereas 61.22% were correctly included in group 2.

Finally, because the wavelengths did not have the same impact score in the classification models, the VIP scores were obtained. Five wavelengths were identified that registered values above the established threshold. So, 1016.08, 1107.06, 1191.76, 1260.78, and 1678.04 nm can be considered as highly influential wavelengths in group discrimination.

In brief, the obtained results reveal how hyperspectral imaging is a promising non-destructive and rapid technology for the classification of beef samples according to their tenderness degree. In future research, in order to test the potential of HSI to classify meat samples according to tenderness, it would be convenient to increase both the number of samples and tenderness groups to be used as input in the classification model.

## Figures and Tables

**Table 1:** Descriptive analysis of the groups established for the case under study.

Analysis	Mean		*p*-Value
	Group 1	Group 2	
WBSF (N)	47.27 ^a^ ± 4.92	64.97 ^b^ ± 11.90	0.000
pH	5.59 ± 0.07	5.59 ± 0.11	0.91
IMF (%)	1.76 ± 0.88	2.09 ± 1.02	0.31
Soluble collagen (mg g^−1^)	0.52 ± 0.26	0.57 ± 0.31	0.50
Total collagen (mg g^−1^)	5.81 ± 1.26	6.37 ± 1.38	0.11

Note: WBSF, Warner Bratzler shear force; IMF, Intramuscular Fat. ^a, b^ Different superscript letters indicate a statistical difference (*p* ≤ 0.05) between the values within the same row. Group 1 (n = 30), the samples with WBSF < 53 N; and group 2 (n = 28), the samples with WBSF > 53 N. Mean ± standard deviation values.

**Table 2:** Rotated matrix with the contribution of each principal component to every single variable.

Analysis	PC1	PC2	PC3	PC4	PC5
pH	−0.035	0.496	−0.822	0.269	−0.074
Intramuscular fat	−0.063	−0.763	−0.515	−0.064	0.381
Total collagen	0.583	−0.113	−0.214	−0.579	−0.516
Soluble collagen	0.619	0.275	0.065	−0.101	0.725
WBSF	0.522	−0.289	0.094	0.760	−0.239

**Table 3:** Confusion matrix for the 3 PLS-DA models selected.

Pre-Processing Algorithms				Real Group (%)	
				1	2
1st der + MC	Estimated group (%)	CV	1	74.58	32.91
			2	25.43	67.09
		Pred	1	73.05	38.78
			2	26.95	61.22
Smoothing + 2nd der + MC	Estimated group (%)	CV	1	72.73	33.43
			2	27.27	66.57
		Pred	1	74.67	42.11
			2	25.33	57.89
Smoothing + SNV + MC	Estimated group (%)	CV	1	72.82	32.60
			2	27.18	67.40
		Pred	1	73.91	45.21
			2	26.09	54.79

Note: 1st der, 1st derivative; MC, mean center; 2nd der, 2nd derivative; CV, cross validation; Pred, prediction.

**Table 4:** Sensitivity and specificity values for the 3 classification models.

		Sensitivity		Specificity	
		CV	Pred	CV	Pred
1st der + MC	1	0.746	0.731	0.671	0.612
	2	0.671	0.612	0.746	0.731
	Average	0.708	0.671	0.708	0.671
Smoothing + 2nd der + MC	1	0.727	0.747	0.666	0.579
	2	0.666	0.579	0.727	0.747
	Average	0.696	0.663	0.697	0.663
Smoothing + SNV + MC	1	0.728	0.739	0.674	0.548
	2	0.674	0.548	0.728	0.739
	Average	0.701	0.643	0.701	0.643

## Data Availability

The data presented in this study are available on request from the corresponding author.
